# Effects of Salt and Homogenization Processing on the Gastrointestinal Fate of Micro/Nano-Sized Colloidal Particles in Bigeye Tuna (*Thunnus obesusis*) Head Soup: *In vitro* Digestion Study

**DOI:** 10.3389/fnut.2022.833712

**Published:** 2022-02-11

**Authors:** Liu Lin, Zhenhai Cao, Xuebing Zhang, Ming Kang, Xichang Wang, Jian Zhong, ChangHua Xu, Long Zhang, Ningping Tao, Shanggui Deng

**Affiliations:** ^1^College of Food Science and Technology, Shanghai Ocean University, Shanghai, China; ^2^Shanghai Engineering Research Center of Aquatic-Product Processing and Preservation, Shanghai, China; ^3^College of Food and Pharmacy, Zhejiang Ocean University, Zhoushan, China

**Keywords:** micro/nano-sized colloidal particles, salt, homogenization, gastrointestinal digestion *in vitro*, microstructure characterization, lipid digestion

## Abstract

The effects of condiment (salt) and processing technic (homogenization) on digestion and interfacial properties of micro/nano-sized colloidal particles (MNCPs) in bigeye tuna head soup (BTHS) using simulated gastrointestinal digestion model *in vitro* were investigated. For MNCPs in BTHS, the triglycerides were wrapped with proteins in the form of a ring. After salting, the average particle size of the MNCPs in salted BTHS (SBTHS) decreased compared with BTHS. However, the partial demulsification phenomenon existed, and part of the protein was encapsulated in some MNCPs. After further homogenization, the average particle size of the MNCPs in homogenous SBTHS (HSBTHS) was further decreased based on SBTHS and the MNCP was rearranged, which changed the original membrane structure. After gastrointestinal digestion, adding salt decreased the release of total fatty acids compared with unsalted. But homogenization processing increased the release of total fatty acids in HSBTHS and there was no significant difference (*p* ≥ 0.05) between HSBTHS and BTHS. Thus, the decrease in the release of some fatty acids due to adding salt was compensated by homogenization. Therefore, the changes in composition and microstructure of MNCPs induced by salt and homogenization might contribute to the digestion difference of MNCPs.

## Introduction

Bigeye tuna (*Thunnus obesusis*), known as “Ocean Gold,” has an important position in the aquatic product consumer market due to the excellent value of utilization and edibility (1). Bigeye tuna is mainly eaten in the form of sushi and sashimi, so a larger amount of by-products with high nutritional value will be produced, including heads, tails, and bones (2). The heads, which are rich in proteins, lipids, and mineral elements as well as contain a full range of amino acids, are one of the main by-products of processing. In addition, the eye sockets, containing a large number of n-3 series polyunsaturated fatty acids (PUFAs) and C22:6 (DHA) can account for 30–40% of the total PUFAs, which is a good source of high-quality fatty acids (FAs) (3). Qian et al. (4) found that the bigeye tuna heads (BTHs) were boiled into fish head soups using the traditional method of boiling fish soup, which can effectively use resources and enrich the soup varieties.

Soup, an important form of obtaining nutrients, plays an indispensable role in food culture. But when nutrients are heated, boiled, and extracted, only a limited number of active ingredients can be migrated into the soup from raw materials. So why do we still choose to eat soup? Studies have shown that during boiling, micro/nano-sized colloidal particles (MNCPs) were formed by the interaction of single molecules dissolved into the soup. Additionally, few studies have reported the medicinal value and biological activity of aggregates in the soup (5–9). Among them, the MNCP aggregates in Ge-Gen-Qin-Lian-Tang decoction have been confirmed to have antidiabetic activity (8), and MNCPs of different particle sizes in tuna head soup had anti-oxidation ability (9). Moreover, the digestion behavior of colloidal particles in soup is rarely reported.

The studies have found that Eicosapentaenoic acid (EPA, C20: 5) and docosahexaenoic acid (DHA, C22: 6) in fish soup were mainly distributed in triglycerides (TGs), which were at the center of MNCPs. The membrane of MNCPs was formed by glycosylated molecules, proteins, and phospholipids, which plays an important role in preserving the integrity of MNCPs (10). Meantime, in the course of processing soup into commodities, the addition of condiments (salt) and the influence of processing technic (homogenization) changed the oil-water interface layer naturally formed by MNCPs (11–13), and may affect its digestion behavior in the gastrointestinal tract and bioavailability. Therefore, it is necessary to investigate how MNCPs and the membrane of MNCPs are modified during digestion, and how their composition and structure affect the bioavailability of lipids. Due to the advantages of convenient operation, short cycle, low cost, and no ethical restrictions, *in vitro* digestion model has been widely adopted. Liang et al. (13) and Zhao et al. (14) have investigated the mechanism of milk fat globules structure and interfacial properties changes on the bioaccessibility of milk fat using *in vitro* digestion model. Therefore, the *in vitro* digestion model was chosen in this study, to expediently monitor the physical and chemical changes of lipids during digestion.

Briefly, in this study, the MNCPs microscopic morphology and particle sizes changes in BTH soup (BTHS), salted BTHS (SBTHS), and homogenous SBTHS (HSBTHS) during each digestion stage of the simulated gastrointestinal tract were investigated using an inverted optical microscope and laser particle sizer. Meanwhile, the effects of salt and homogenization on FAs release, distribution, and change regulations of main components in MNCPs were revealed using gas chromatograph (GC), laser scanning confocal microscopy (LSCM), and infrared transmission imaging during gastrointestinal digestion, respectively. Therefore, this study not only uncovered the MNCPs microscopic morphology change regulations and clarified the effects of salt and homogenization on the digestion characteristics of MNCPs, but also offered a foundation for the correlation between the absorption and utilization of MNCPs in the human body and cooking conditions.

## Materials and Methods

### Materials and Reagents

The Bigeye tuna heads (*Thunnus obesusis*, number: 30; weight: 1.58 ± 0.23 kg; length: 26.39 ± 2.54 cm; width: 25.33 ± 1.30 cm) were obtained from Dalian Xiang Xiang Food Co., Ltd. (Dalian, Liaoning, China). The bigeye tunas were captured using a seine net in the Pacific-Indian Ocean. All the heads were frozen before coming to the laboratory and stored at −40°C before use (a maximum of 4 weeks). The mixture of 37 FA methyl esters (FAME), C19:0 (purity 99%), and C19:0 FAME was purchased from Shanghai ANPEL Scientific Instrument Co., Ltd. (Shanghai, China). Methanolic, chloroform, boron trifluoride-methanol (14% in methanol), and n-hexane of high-performance liquid chromatography (HPLC) grade; KCl, KH_2_PO_4_, NaHCO_3_, NaCl, MgCl(H_2_O)_6_, (NH4)_2_CO_3_, CaCl(H_2_O)_2_, NaOH, absolute sodium sulfate and acetone and other analytical reagents were purchased from the Chinese Medicine Chemical Reagent Co., Ltd. (Shanghai, China). Pepsin from porcine gastric mucosa (V900497, 750 U/mg) and pancreatin from porcine pancreas (P7545, 50 U/mg) were purchased from Sigma-Aldrich (St. Louis, MO, USA). The fluorescent dye Nile Red (9-diethylamino-5H-benzoalpha-phenoxazine-5-one), porcine bile salts, phosphate buffered saline (PBS, 0.2M, pH 7.0), and Mcilvaine Buffer (MB, 0.2M, pH 2.5) were bought from Shanghai Macklin Biochemical Co., Ltd. (Shanghai, China). The fluorescent dye Fast Green FCF was obtained from Avanti Polar Lipids, Inc. (Alabaster, Alabama, USA). Distilled and deionized water were purified using a Milli-Q system (Millipore Corp., Billerica, Massachusetts, USA).

### Cooking of Fish Head Soup

The cooking of soup used the method of Qian (4) and Fan (11) with some modification. The BTHS was prepared as follows: the frozen BTHs were thawed using running water before removing the gills. The heads were cut into small pieces of ~3 × 3 × 2 cm^3^ using a cleaver, washed, and drained. Soybean oil (20 ± 1 g, Yihai Kerry Food Marketing Co., Ltd., Shanghai, China) was poured into the pot and heated at 120°C for 30 s in an induction cooker (C21-WT2112T, Midea Co. Ltd., Foshan, Guangdong, China), and subsequently, the headpieces (F) were fried at 120°C for 40 s. The fried fish headpieces and 3.2 kg drinking water (1:8, w/w) were cooked at 97 ± 2°C for 30 min, and then at 90 ± 2°C for 120 min. 0.5% (salt/water, w/w) salt (300 g/pack, China National Salt Industry Group Co., Ltd., Beijing, China) was added to BTHS to prepare SBTHS. SBCHS (1L) was cooled to room temperature (25 ± 2°C) and homogenized (10–20 MPa, 2 min, 25±2°C) using a high-pressure homogenizer (GYB60-6S, Shanghai Donghua High Pressure Yunjiang Pump Factory, Shanghai, China), repeated two times to prepare HSBTHS. The BTHS, SBTHS, and HSBTHS were collected and stored at −40°C before use.

### Gastrointestinal Digestion

The simulated gastric fluid (SGF) and simulated intestinal fluid (SIF) were made up of the corresponding electrolyte stock solutions (Table 1) as previously described by Minekus et al. (15).

**Table 1:** Preparation of simulated digestion fluids.

			**SGF**		**SIF**	
**Constituent**	**Concentration**		**Volume of stock**	**Concentration in SGF**	**Volume of stock**	**Concentration in SIF**
	g/L	mol/L	mL	mmol/L	mL	mmol/L
KCl	37.3	0.5	6.9	6.9	6.8	6.8
KH_2_PO_4_	68.0	0.5	0.9	0.9	0.8	0.8
NaHCO_3_	84.0	1.0	12.5	25.0	42.5	85.0
NaCl	117.0	2.0	11.8	47.2	9.6	38.4
MgCl_2_(H_2_O)_6_	30.5	0.15	0.4	0.1	1.1	0.33
(NH4)_2_CO_3_	48.0	0.5	0.5	0.5	-	-
CaCl_2_(H_2_O)_2_	44.1	0.3	-	0.15	-	0.6

*The final volumes of simulated gastric fluid (SGF) and simulated intestinal fluid (SIF) are both 500 ml. It is recommended that the stock solution should be prepared into a concentrated solution (× 1.25, 400 ml)with deionized water and stored at −20°C for later use. CaCl_2_(H_2_O)_2_ is added before digestion to prevent Ca^2+^ from forming precipitation*.

#### Gastric Digestion

The *in vitro* gastric digestion model used the method of Minekus et al. (15) and was modified as appropriate. Different soup samples (100 ml) were mixed with SGF (50 ml). The mixture was acidified to pH 2.0 using HCl (6 M) and incubated for 10 min at 37°C in a constant temperature shaking water bath (SW22, JULABO Corp., Seelbach, Rheinland-Pfalz, Germany) at 120 rpm. Then, 0.3 g pepsin was added and the mixture was incubated in the constant temperature shaking water bath for 2 h (37°C, 120 rpm). The samples were collected periodically for further characterization.

#### Intestinal Digestion

The *in vitro* intestinal digestion model used the method of Minekus et al. (15) and was modified as appropriate. The different gastric samples-chyme (150 ml) was mixed with SIF (150 ml). The mixture was acidified to pH 7.0 using NaOH (1 M). Then the mixture was mixed with 0.6 g pancreatin and 1.28 g porcine bile salts. The mixture was incubated in the constant temperature shaking water bath for 2 h (37°C, 120 rpm) and the pH was maintained at 7.0 ± 0.1 using NaOH (1 M). The samples were collected periodically for further characterization.

### Particle Size and Particle Size Distribution Range

The particle size and particle size distribution range of MNCPs in different samples were determined using the method of Zhang et al. (16) and were modified as appropriate. The sample was diluted to a suitable shading range and then measured using a laser particle sizer (MS3000, Malvern Instruments Co., Ltd., Malvern, UK) at room temperature. Among them, the shading ranges of the different intestinal samples-chyme were 5–15% and others were 5–10%. The undigested and intestinal samples-chyme were diluted using PBS. The gastric samples-chyme were diluted using MB. The refractive index of MNCPs and dispersed phase were 1.460 and 1.330, respectively. The average particle size was expressed using D (2, 3) (surface area average particle size).

### Optical Microscopy Measurements

Briefly, 15 μl sample was placed on a glass slide (4951PLUS-001E, Thermo Fisher Inc., Waltham, Massachusetts, USA) and a square cover glass (10212020C, 20 × 20 mm, Citotest Labware Manufacturing Co., Ltd, Haimen, Jiangsu, China) was covered on the dyed sample and observed using an inverted optical microscope (Shanghai Mingzi Precision Instrument Co., Ltd., Shanghai, China).

### LSCM Measurements

The sample was prepared using the method of Lin et al. (10). Briefly, 1 ml sample was mixed with 100 μl Nile red staining solution (42 μg/ml in acetone) and 10 μl fast green FCF staining solution (1 mg/ml in water) to dye the TGs and protein, respectively. The dyed sample (10 μl) was placed on a glass slide (4951PLUS-001E, Thermo Fisher Inc., Waltham, Massachusetts, USA) and a square cover glass (10212020C, 20 × 20 mm, Citotest Labware Manufacturing Co., Ltd, Haimen, Jiangsu, China) was covered on the dyed sample and observed using an LSCM (TCS SP8, Leica Instruments Co., Ltd., Wetzlar, Hesse, Germany). The dye preparation and the dyeing were protected from light to prevent fluorescent dye quenching.

An argon laser operating at 488 nm excitation wavelength (detection of emission between 500 and 535 nm), and a He-Ne laser operating at 543 nm excitation wavelength (detection of emission between 565 and 615 nm) were used for confocal experiments with a 63 x 1.4 oil immersion objective. The images were processed and measured using Zeiss LSM Image Browser off-line software (Carl Zeiss Corp., Oberkochen, Baden-Württemberg, Germany).

### Infrared Microscopy Imaging

The sample was prepared using the method of Yao et al. (17) and was modified as appropriate. Briefly, 7 μl sample was placed on a ZnSe window (13 × 2 mm, Crystal GmbH, Berlin, Germany), then the ZnSe window was kept at room temperature for 30 min, air-dried, and analyzed using a Fourier transform infrared spectroscopy Spectrum Frontier/Spotlight 400 Microscopy System (PerkinElmer, Inc., Waltham, Massachusetts, USA). The scanning conditions were: infrared light absorption map size: 400 × 400 μm, scanning wave number range: 4,000–750 cm^−1^, resolution: 8 cm^−1^, and image pixel: 6.25 cm^−1^. The images that were utilized to analyze were obtained after the removal of atmospheric noise and baseline correction using Spectrum software (PerkinElmer, Inc., Waltham, Massachusetts, USA).

### Fatty Acids

#### Extraction of Total Lipids

The total lipids were extracted using the method of Folch et al. (18). Briefly, ~20 ml fish head soup and 400 ml chloroform:methanol (2:1, v/v) were kept at 4°C for 24 h. The solution was filtered using filter paper (No. 101, ϕ15 cm, Beimu Pulp Paper Co., Ltd., Hangzhou, Zhejiang, China), and NaCl solution (0.9%, w/w) was added and kept at 4°C for 3 h. Then the upper methanol phase was removed using a 50 ml disposable syringe (Sheng Bei Nuo Medical Supplies Co., Ltd., Shanghai, China). The chloroform was removed using a rotary evaporator (R250, Buchi Laboratory Equipment Trading (Shanghai) Ltd., Shanghai, China) at 40°C. The remaining material was the total lipids.

#### Analysis of FAs Composition

The total lipids were converted to FAME derivatives using the method of Lin et al. (10) and Zhang et al. (19). The total lipids (~0.10 g) was mixed with 100 μl C19:0 internal standard (10 mg/ml) and 5 ml methanolic-NaOH (0.5 mM). The mixture was put into a round-bottomed flask and connected with a reflux condenser before placing in a thermostat water bath (HWS-24, Shanghai Yiheng Scientific Instruments Co., Ltd., Shanghai, China), shaken at 100°C for 10 min. Next, 3 ml 14% boron trifluoride in methanol was added and shaken at 100°C for 5 min, and 2 ml n-hexane was added and shaken at 100°C for 2 min, and 10 ml saturated NaCl solution was added. The reflux condenser was connected to running water, and its top was plugged with degreasing cotton (500 g/bag, Wucheng Wenjiang Sanitary Material Co., Ltd., Dezhou, Shandong, China) to prevent the FAME derivatives from volatilizing. The mixture was held for 5 min, and the upper layer was collected using a 2 ml disposable syringe (Sheng Bei Nuo Medical Supplies). The solution was filtered using a nylon syringe filter (13 mm × 0.22 μm, Shanghai Anpel Scientific Instrument) and injected in a 2 ml injection bottle (32 × 11.6 mm, Shanghai Anpel Scientific Instrument) for further FA composition analysis using a GC.

The GC (TRACE GC, Thermo Fisher Inc., Waltham, Massachusetts, USA) was equipped with an Agilent SP-2560 capillary column (100 m × 0.25 × 0.2 μm, Agilent Technologies Inc., Palo Alto, California, USA) and a flame ionization detector (Thermo Fisher). Nitrogen was used as the carrier gas at a flow rate of 1 ml/min. The initial column temperature was 70°C, raised to 140°C (20°C/min), and held for 1 min; then raised to 180°C (4°C/min) and held for 1 min; then raised to 225°C (3°C/min) and held for 30 min. The gasifying temperature was 250°C. The injection volume was 1 μl with a split ratio of 45:1. The FAME was identified by comparison with the retention times of the standard FAME mixture. The FAs were determined using the area ratio of the GC peak between internal standard C19:0 and different FAME standards.

#### Calculation of FA Release Rate

The rate of the released FA after gastrointestinal digestion = (FA of undigested soup - FA of soup after gastrointestinal digestion)/ FA of undigested soup × 100%.

### Statistical Analysis

All the above experiments were repeated three times and each sample was repeated two times. All the values were from independent triplicates expressed as mean ± SD. The data were compared using one-way ANOVA using the Statistical Package for the Social Sciences 16.0 software (SPSS Institute, Inc., Chicago, Illinois, USA). A value of *P* < 0.05 was considered to have significant differences between the data. Origin 8.0 (Origin Lab, Northampton, Massachusetts, USA) was used to process and generate images.

## Results And Discussions

### Effects of Salt and Homogenization on the Particle Size and Distribution of MNCPs During Gastrointestinal Digestion

**Figure 1:**
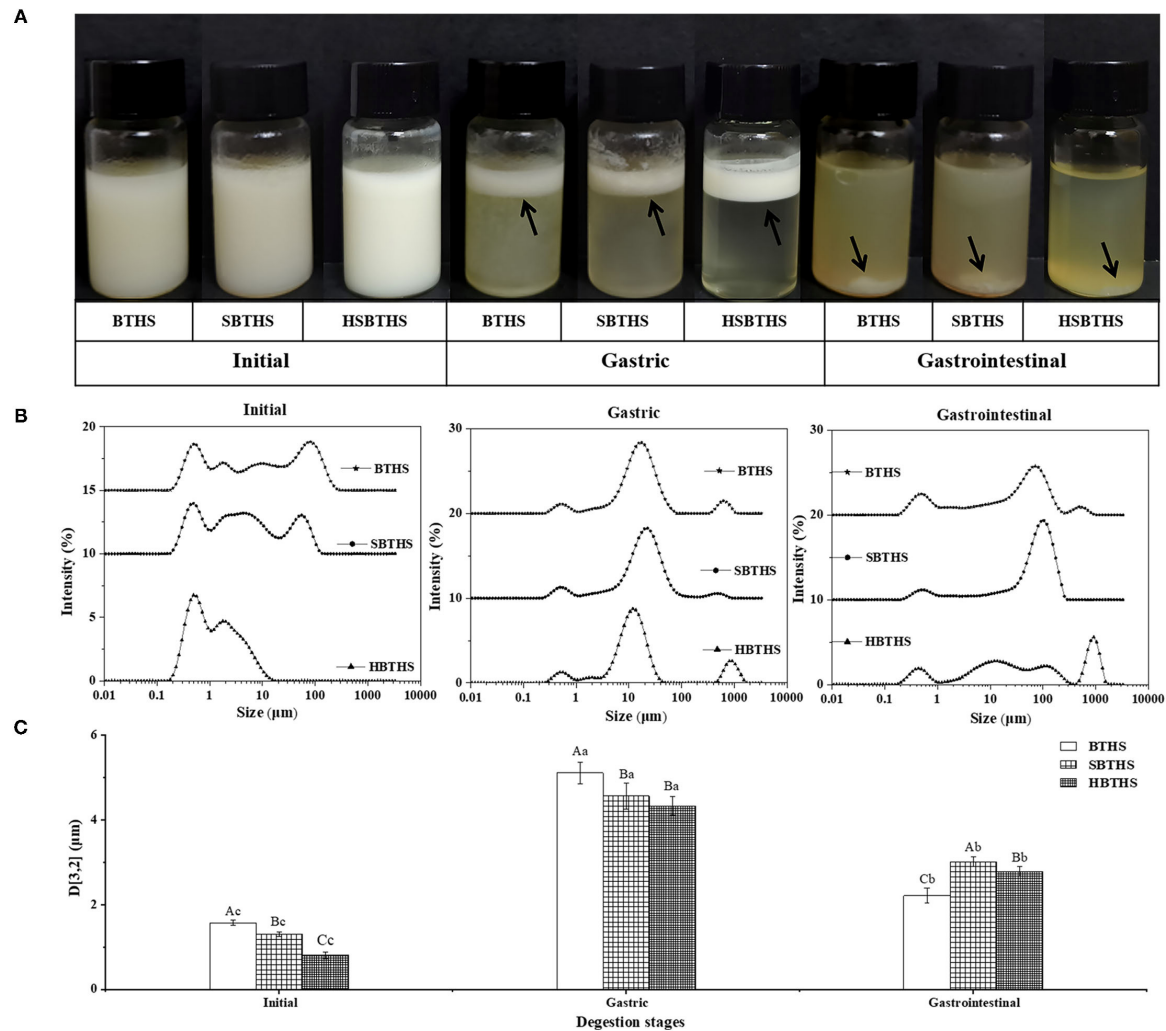
The photos **(A)**, the surface-weighted mean particle diameter **(D[3,2],B)** and the particle size distributions **(C)** of digestion product of BTHS, SBTHS and HSBTHS in each digestion stage of the simulated gastrointestinal tract. Lowercase letters indicate the difference of different digestion stages of the same soup, uppercase letters indicate the difference of the same digestion stage of different soups. Different letters indicate a significant difference (*P* < 0.05). Data are the mean ± SD (*n* = 3).

After digestion of BTHS, SBTHS, and HSBTHS by the gastrointestinal tract, the figures of digestive products (Figure 1A), average particle size (Figure 1C), and particle size distribution (Figure 1B) at each stage are shown in Figure 1. As shown in Figure 1, the appearance of the fish soup is not changed significantly after adding salt and homogenizing. At the initial stage, three kinds of fish soup showed uniform and stable milky white liquid. It can be seen from the initial stage of Figure 1B that MNCPs are ranging from nano- to micro-scale in all soups. Among them, both BTHS and SBTHS exhibited obvious multi-peak distribution in the initial stage. However, it could be noted that compared with BTHS, SBTHS had a lower peak height on the right side and the peak shifted to the left as a whole. With the average particle size in Figure 1C, it can be seen that the particle size of MNCPs in SBTHS is decreased by 0.27 μm with reasons that NaCl improves the hydration of proteins, enhances the combination between proteins and between proteins and fats, and promotes the emulsification of lipid droplets in fish soup (20). After further homogenization of the fish soup, the average particle size of HBCHS reduced to 0.799 μm, and its particle size distribution was more concentrated than that before homogenization but did not show a unimodal distribution as well. The reason may be that after high-pressure homogenization, the original stable structure of MNCPs in the soup was destroyed and rearranged, while the protein in the soup was not enough to wrap all the fine MNCPs formed by shearing, resulting in adjacent MNCPs having greater attraction than the repulsion between them, and then they gathered.

After gastric digestion, obvious phase separation occurred in all soups (Figure 1A), mainly consisting of oil layer at the top, fine and soft flocculent layer in the middle, and water phase accompanied by a small number of floccules at the bottom. The particle size distribution in all soups gradually changed from the original multi-peak distribution to the single-peak normal distribution (Figure 1B), and the average particle size increased remarkably (*P* < 0.05, Figure 1C), which indicated that MNCPs had obvious aggregation behavior. These results were consistent with the changes of milk fat globules in bovine milk (13).

This was because pepsin played a role in digesting part of the protein on the MNCPs membrane so that the original stable structure of MNCPs was changed, the repulsive force between MNCPs was reduced, resulting in aggregation between colloidal particles. In addition, acidic conditions in the gastric changed the pH of the original system in the soup, causing the flocculation of incompletely digested protein. The same as the initial stage, the peak shapes of BTHS and SBTHS were more similar after gastric digestion.

After gastrointestinal digestion, the floccules disappeared, forming insoluble complexes visible to the naked eye in the soup (Figure 1A). It can also be seen from Figure 1B that the particle size of MNCPs increases remarkably, and its peak shifts to the right. The significant reduction of colloidal particles in the original particle size range might indicate that most of the MNCPs in the soup had been digested, producing colloidal particles with large particle size (100–1,000 μm), which might be insoluble bile salt complex produced during intestinal digestion or calcium soap formed by combining Ca^2+^ in the digestive liquid with FAs released during the intestine digestion. Generally speaking, the changing trends of MNCPs in all soups were similar, with the average particle size increasing first decreasing then and being larger than that before digestion (Figure 1C). It could be concluded from the specific values that there were differences in the changes of digestion of MNCPs in SBTHS and HSBTHS. The effects of adding salt and homogenization on the digestion of MNCPs in the soup still need to be further analyzed in combination with its morphological changes.

### Effects of Salt and Homogenization on the Morphological Characteristics of MNCPs During Gastrointestinal Digestion

**Figure 2:**
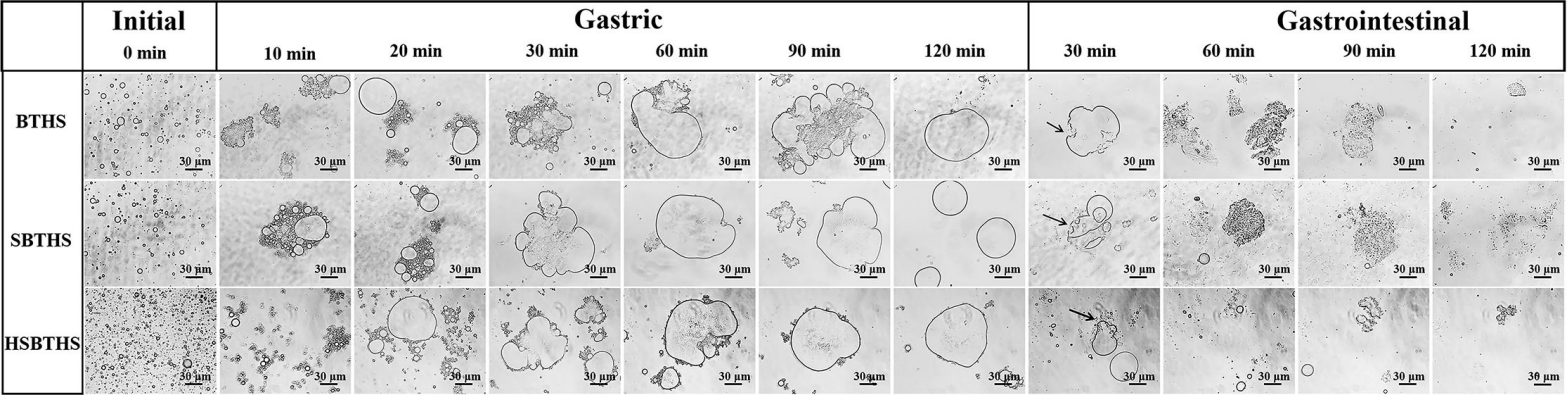
The optical pictures of MNCPs in BTHS, SBTHS, and HSBTHS during each digestion stage of the simulated gastrointestinal tract. Scale bar = 30 μm.

The morphological changes of MNCPs in BTHS, SBTHS, and HSBTHS during gastric digestion are shown in Figure 2. In the initial stage, spherical colloids with different sizes and complete edges could be observed, and they were evenly dispersed in the soup. Among them, the amount of MNCP in HSBTHS increased markedly and its size decreased significantly, which was consistent with the results of particle size measurement. During gastric digestion, the MNCP mainly exhibited aggregation behavior, that is, the aggregation process of lipid droplets. According to the previous research results in the laboratory, the MNCP in the soup was a lipid droplet with TG as its core and coated with nutrients such as protein, phospholipid, and sugar (10). Therefore, the aggregation of MNCPs was mainly attributed to the decomposition of membrane proteins by pepsin into peptone during gastric digestion, which damaged the originally stable MNCPs membrane, causing TGs to be exposed, and colloidal particles to aggregate. It was also attributed to the simulated acidic environment in SGF and high ion concentration (21, 22). As could be seen from the gastric stage (Figure 2), the morphology of MNCPs is different in different digestion nodes, especially in HSBTHS. The colloidal particles changed most greatly in the first 10 min of the whole process of gastric digestion, with MNCPs moving closer to each other, lipid droplets merging, and colloidal particles becoming larger. When it came to the 10 min, complete spherical colloidal particles could not be found anymore in the center of some MNCPs aggregates in BTHS, but the edges of MNCPs aggregates were still uneven, while they could still be found in the aggregates of SBTHS, whose reason was that the Na^+^ in SBTHS was distributed on the surface of MNCPs membrane and the stability of MNCPs membrane was further improved because of the change of substance composition on the MNCPs membrane, thereby changing the digestion behavior (11, 12). The obvious difference between BTHS and SBTHS was that MNCPs in HBCHS also aggregated but existed in the form of small colloidal particles instead of large lipid droplets.

In the following process of digestion, the colloidal particles continued to merge. When it came to 20 min, large-diameter spherical colloidal particles with complete edges could be found in BTHS, while there was no obvious change was found in SBTHS. When it came to 30 min, MNCPs with the same particle size as that in the initial stage could not be found in the center of aggregates in SBTHS, but the edges of aggregates were still uneven and not completely fused. After digestion for 60 min, there was no obvious change in the morphology of lipid droplets in BTHS and SBTHS, and at the end of gastric digestion, aggregates became smooth and intact. Compared with BTHS and SBTHS, although HSBTHS also produced large lipid droplets, there were still a wide range of fine colloidal particles around the aggregates or existing alone. This was because homogenization broke the lipid droplets, and the original protein on the membrane was not enough to cover all the newly formed MNCPs. Therefore, the dispersed proteins and surfactant active substances in the soup were re-adsorbed to the interface to make up a new membrane structure, increasing the stability of colloidal particles.

The morphological changes of MNCPs in BTHS, SBTHS, and HSBTHS during intestinal digestion are shown in Figure 2. In the whole process of intestinal digestion, the membrane structure of MNCPs aggregates was completely destroyed under the action of trypsin, which was regarded as the process of cleavage of MNCPs aggregates. When it came to 30 min, the colloidal particles in BTHS and SBTHS became irregular and the edges began to rupture, as indicated by the arrows. After 60 min, the surface tension of lipid droplets was further lowered under the action of bile salt, and obvious emulsification occurred to them and fine droplets were formed. However, these droplets still existed in clump instead of spreading out. At the end of digestion, most of the lipid droplets disappeared, but larger aggregates and sporadic small colloidal particles could still be found. Those aggregates might be lipid droplets that were not digested completely or lipid-containing aggregates formed by bile salts and phospholipids, monoglycerides, or FAs.

### Effects of Salt and Homogenization on the Microstructure Changes of MNCPs During Gastrointestinal Digestion

**Figure 3:**
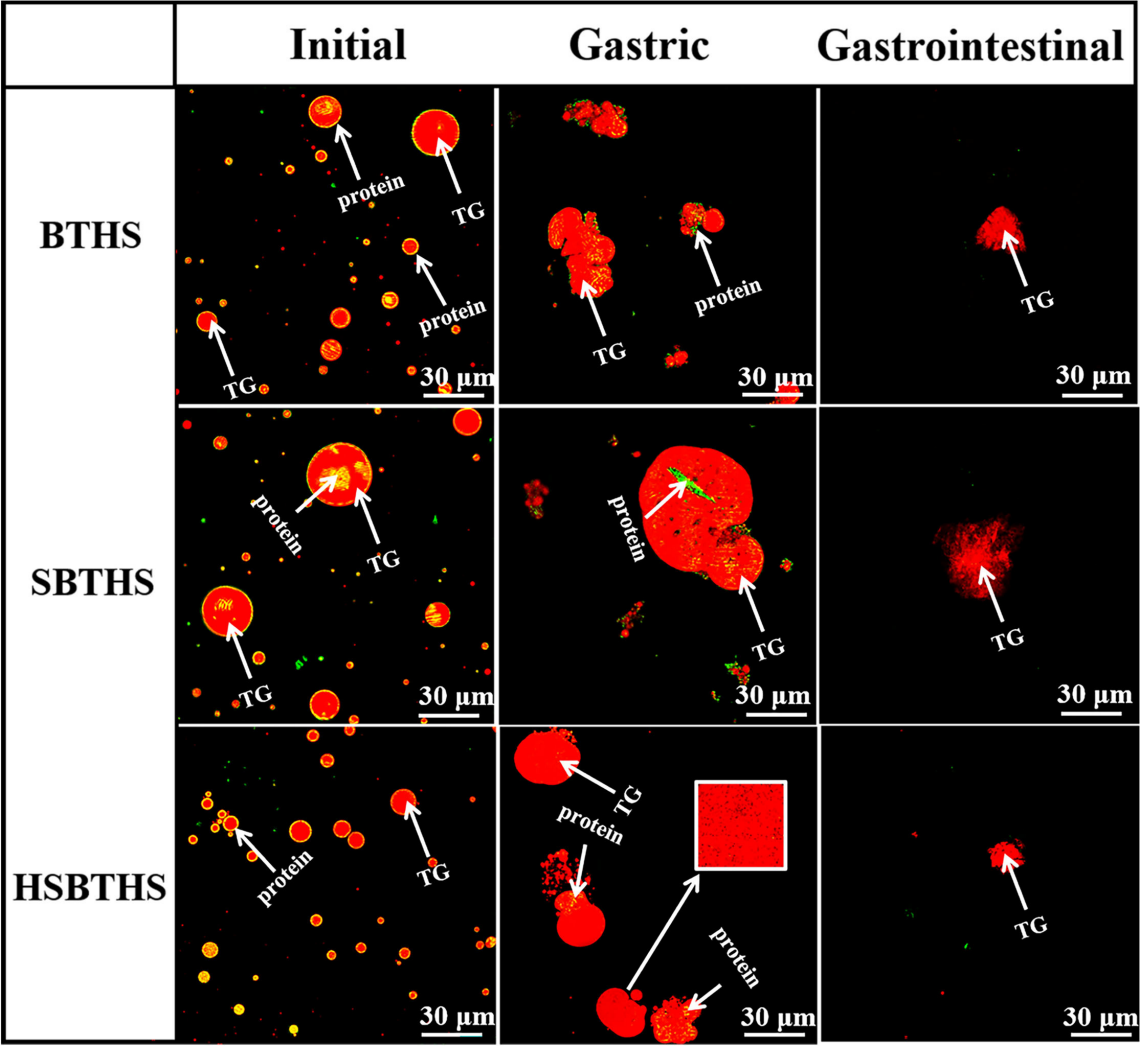
The laser confocal images of MNCPs in BTHS, SBTHS, and HSBTHS during each digestion stage of the simulated gastrointestinal tract. Scale bar = 30 μm.

To further analyze the changes of nutrients in MNCPs in the process of gastrointestinal digestion, and verify the results of optical microscopy, Nile red and fast green staining solution were used to stain TGs and protein in MNCPs, respectively, and then LSCM was used to observe the microstructural changes of MNCPs in all soups during gastrointestinal digestion. As shown in Figure 3, at the initial stage, the TGs marked by the red fluorescent probe are wrapped with protein marked by the green fluorescent probe, which is consistent with former literature reports (10). In SBTHS, the particle size of some MNCPs became larger, and protein could be clearly observed in the MNCPs, which is possibly due to the local salt concentration being high, NaCl made the colloidal particles adsorb each other to form colloidal particles with a large particle size by decreasing the electrostatic repulsion on the colloidal surface, wrapping the water-soluble proteins in the soup or the proteins on MNCP membrane. After being physically broken, the size of the MNCP that could be observed in HSBTHS was smaller and more widely distributed, which was consistent with the results of the particle size and optical microscopy.

After gastric digestion, the protein clots split; the rings disappeared; the fat globules were released; and the TG wrapped in the center of MNCP was released and aggregated to form TG aggregates. It was noteworthy that spherical colloidal particles with intact structure could be observed after gastric digestion. Similarly, after milk fat globules were digested by the gastric, the structure of some milk fat globules was still intact. It was found that the phospholipids coated on the periphery of milk fat globules were not digested, which still wrapped the lipid droplets in a ring, so that the structure of fat globules remained stable without obvious changes, playing an important role in protecting the integrity of milk fat globules (23). Previous studies in our laboratory have shown that there were also phospholipids on the membrane of MNCPs in the fish soup (4, 10, 11). Therefore, phospholipids and sugars on the membrane of MNCPs played an important role in protecting the integrity of MNCPs during gastric digestion. Moreover, the gastric digestion products of the three fish soups were undigested protein and part of the protein that transferred from the original ring-shaped distribution on the periphery of the MNCPs to the center of the lipid droplet (as shown by the arrow). There were three main reasons for the incomplete degradation of proteins. First, some proteins could not be decomposed by pepsin due to their high glycosylation, and another part proteins were not degraded by pepsin, and they needed to be further degraded by trypsin in the small intestine (24–26). Second, after gastric digestion, the lipid droplets were rearranged and aggregated, and in the process of aggregation, the protein was wrapped inside the lipid droplets, blocking the contact between protein and pepsin. Third, as the pH fluctuated during digestion, the activity of pepsin was reduced, thus weakening its enzymolysis ability. Meanwhile, it could be noticed that there were many black holes in the center of the lipid droplets of SBTHS and HSBTHS, and smaller holes in HSBTHS, as shown in the box. It was found in early studies (11) that after adding salt, Cl^−^ combined with TGs and formed black holes during digestion. Two possible reasons for that were Cl^−^ hindered the fusion of TGs during digestion, or vesicles were formed by undigested phospholipids.

When the digestive products from the gastric entered into the intestinal digestion, bile salt was attached around the lipid droplets to replace the phospholipids and proteins on the membrane of MNCPs, turning the fat globules into microemulsions, increasing the contact area between the lipid droplets and pancreatic lipase and colipase to promote lipid digestion. At the same time, the undigested proteins in gastric digestion were further hydrolyzed under the influence of trypsin, with no green fluorescence region being obviously observed. After intestinal digestion, there were still red fluorescent regions with black holes in the digestive products, but their edges were blurred, which might come from incomplete digested MNCPs. It may also come from fat-containing floccules formed by bile salts, phospholipids, lipolysis products, and undegraded TGs, which were distinguished from the original MNCPs. It could be observed that there were great differences in the size and dispersion degree of fat droplets in the three fish soups after simulated gastrointestinal digestion.

### Effects of Salt and Homogenization on the Spatial Distribution of MNCPs During Gastrointestinal Digestion

**Figure 4:**
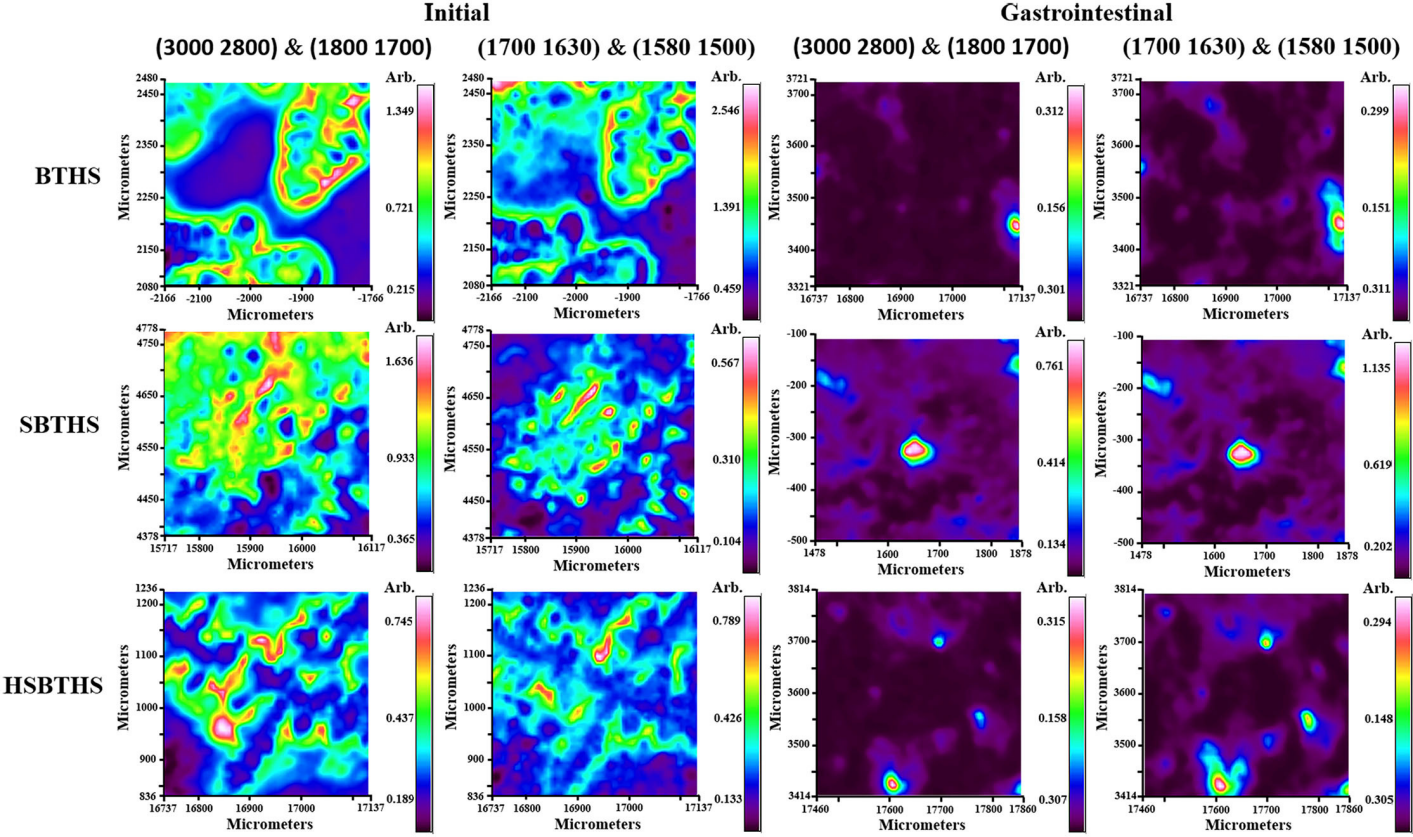
The infrared spectroscopic images of MNCPs in BTHS, SBTHS, and HSBTHS before and after gastrointestinal digestion.

The chemical composition and spatial distribution information of the sample in a certain micro-area can be obtained using infrared microscopy imaging technology (27). The infrared spectroscopic images with different digestion stages (initial and after gastrointestinal digestion) of BTHS, SBTHS, and HSBTHS are shown in Figure 4, which were based on the main components of soup (lipids and proteins).

The wave-number segments (3,000, 2,800 cm^−1^) and (1,800, 1,700 cm^−1^) were chosen to represent the lipids in all samples. For the initial stage, the red pixels areas could be clearly observed in all soups, indicating that soups are high in lipids, which was consistent with broth (17). But they differ from the results of optical microscope and LSCM in which lipids existed individually in the form of MNCPs, and most lipids existed in the form of MNP aggregates in the infrared spectroscopic images. The reason may be that the osmotic pressure of the sample was changed by air-drying, inducing the MNP combined to form larger aggregates. The areas of red pixels were significantly reduced and existed in the form of spherical particles after gastrointestinal digestion. Meanwhile, the area of the red pixel was the largest in SBTHS, and the area of red pixels in HSBTHS was close to that in BTHS. These results indicate that most lipids have been digested after gastrointestinal digestion, and the digestion degree of HSBTHS was similar to that of BTHS and better than SBTHS.

The wave-number segments (1,700, 1,630 cm^−1^) and (1,580, 1,500 cm^−1^) were chosen to represent the protein in all samples. For the initial stage, the red pixels areas that represented the proteins were less than that of lipids, indicating that the proteins were less than the lipids in all soups, which were consistent with Qian et al. (4) and Fan et al. (11). A small number of red pixel areas that represented the proteins were contained in all soups after gastrointestinal digestion. These results indicate that a small number of proteins have been undigested after gastrointestinal digestion, which was consistent with the results of LSCM.

### Effects of Salt and Homogenization on the Release of FAs of MNCPs After Gastrointestinal Digestion

**Figure 5:**
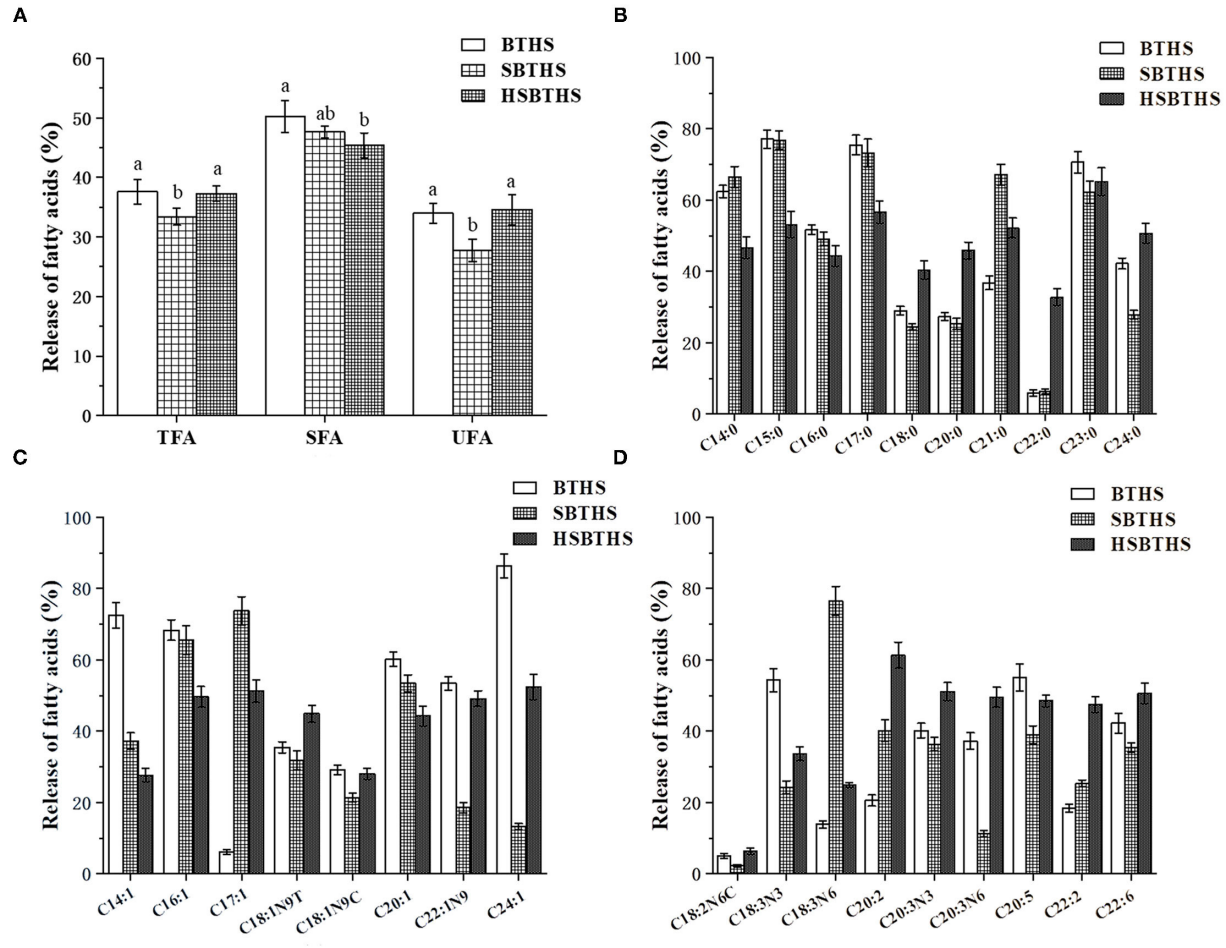
The release ratio of different types FAs [w/w, %, **(A)**], saturated FAs [SFAs, w/w, %, **(B)**], monounsaturated FAs [MFAs, w/w, %, **(C)**], and PUFAs [w/w, %, **(D)**] of MNCPs in BTHS, SBTHS and HSBTHS after gastrointestinal digestion. TFA, SFA, and UFA indicate total FAs, saturated FAs, and unsaturated FAs, respectively. Lowercase letters indicate the difference of the same FAs of different soups. Different letters indicate a significant difference (*P* < 0.05). Data are the mean ± SD (*n* = 3).

The FAs composition before and after the digestion of MNCPs in the three soups were analyzed, and the release rate of saturated FAs (SFAs, Figure 5B), monounsaturated FAs (MFAs, Figure 5C), and PUFAs (Figure 5D) were calculated, which are shown in Figure 5. As shown in Figure 5A, the total FAs (TFAs) release rate of SBTHS was 33.39%, which was significantly lower (*P* < 0.05) than that of BTHS, which was 37.58%. The TFAs release rate of SBTHS was decreased by 4.19% compared with BTHS. The reason may be that the stability of MNCPs was increased by the addition of Na^+^ and Cl^−^ (11), and the formation of insoluble soaps was promoted by the increased Na^+^ concentration, which reduced the release of FAs compared with BTHS. The TFAs release rate of HSBTHS was increased by ~3.90% compared with SBTHS, and there was no significant difference (*p* ≥ 0.05) between HSBTHS and BTHS. It can be seen that the reduction of the release rate of some FAs in SBTHS can be made up by homogenization.

In Figures 5B–D, there are 27 FAs released in all soups, including 10 SFAs, 8 MUFAs, and 9 PUFAs, but it can be seen from the specific values that the release rate of each FA is different. C20:5 (EPA) and C22:6 (DHA) are two important N-3 series PUFAs, which have important physiological effects on human health. It was worth noting that the release rate of EPA and DHA in SBTHS was significantly lower (*P* < 0.05) than that in BTHS, but the release rate of them in HSBTHS were increased by ~9.46% and ~15.12% compared with SBTHS, among which the release rate of DHA was even ~8.28% higher than that of BTHS. The reason may be that the binding sites of enzymes and lipids were increased by homogenization, promoting the release of FAs. Thus, the decrease in the release of FAs due to the addition of salt was compensated by homogenization.

## Conclusion

In this study, the digestive characteristics of MNCPs and the effects of salt and homogenization on their digestive characteristics were uncovered from the perspectives of the characterization of digested products and the release of FAs using simulated gastrointestinal digestion model *in vitro*. The results showed that the overall trend of the MNCPs in the three soups was similar in the process of the gastrointestinal digestion tract. In gastric digestion, the protein on the MNCPs membrane was degraded, and the particle size was increased with the fusion between the MNCPs, which was the aggregation behavior of the MNCPs. During intestinal digestion, lipid droplets were dispersed by the effect of bile salt and pancreatin, and MNCPs aggregates were cleaved. After the salt was added, the average particle sizes of MNCPs in SBTHS decreased, but the local demulsification phenomenon existed, which made the MNCPs adsorb and aggregate with each other, and part of the protein was encapsulated in it. At the same time, the stability of MNCPs was increased, and thus the rate of TFAs release was reduced by ~4.19%. After further homogenization, the particle size of the MNCPs in HSBTHS was further decreased and the MNCPs were rearranged, which changed the original membrane structure, increased the contact sites between lipids and enzymes in MNCPs, and promoted the release of TFAs. The TFA release rate of HSBTHS was increased by ~3.90% compared with SBTHS, and there was no significant difference (*p* ≥ 0.05) between HSBTHS and BTHS. Among them, the release of DHA in HSBTHS was increased by ~15.12% compared to SBTHS and even ~8.28% higher than that of BTHS. In summary, the destruction of the stable membrane structure at the interface of MNCPs was the prerequisite for the digestion of MNCPs, and the homogenization to some extent compensated for the reduction in the release of some FAs after adding salt. In the commercialization process of soup, it is necessary to further improve the quality of soup by homogenization, which is contributed to the absorption of nutrients in the soup in the human body.

## Data Availability Statement

The original contributions presented in the study are included in the article, further inquiries can be directed to the corresponding author.

## Author Contributions

LL was involved in the conceptualization, methodology, conducting the experiment, and writing and editing of the original manuscript. ZC contributed to methodology, conducting the experiment, and data curation. XZ and MK conducted the experiment. XW contributed to supervision and project administration. JZ and CX contributed to the methodology. LZ reviewed and edited the manuscript. NT was involved in the conceptualization, methodology, and review and editing of the manuscript. SD provided resources. All authors contributed to the article and approved the submitted version.

## Funding

This study was supported by the National Key R&D Program of China (Project No. 2020YFD0900905).

## Conflict of Interest

The authors declare that the research was conducted in the absence of any commercial or financial relationships that could be construed as a potential conflict of interest.

## Publisher's Note

All claims expressed in this article are solely those of the authors and do not necessarily represent those of their affiliated organizations, or those of the publisher, the editors and the reviewers. Any product that may be evaluated in this article, or claim that may be made by its manufacturer, is not guaranteed or endorsed by the publisher.

## References

[1] Wang X Xie J Chen X. Differences in lipid composition of Bigeye tuna (*Thunnus obesus*) during storage at 0°C and 4°C. Food Res Int. (2021) 143:110233. 10.1016/j.foodres.2021.110233 33992346

[2] Herpandi N Rosma A Wan Nadiah W. The tuna fishing industry: a new outlook on fish protein hydrolysates. Compr Rev Food Sci F. (2011) 10:195–207. 10.1111/j.1541-4337.2011.00155.x

[3] Zhou Y Li Y Dai Z Su F. Analysis and evaluation on nutrition components of three kinds of tuna. Farm Products Process. (2018) 5:43–7.

[4] Qian X Fan X Su H Zhang J Tao N Zhong J. Migration of lipid and other components and formation of micro/nano-sized colloidal structure in tuna (*Thunnus obesus*) head soup. LWT. (2019) 111:69–76. 10.1016/j.lwt.2019.04.088

[5] Ke L Zhou J Lu W Gao G Rao P. The power of soups: super-hero or team-work? Trends Food Sci Tech. (2011) 22:492–7. 10.1016/j.tifs.2011.06.004

[6] Le C Su H Tao N. Antioxidant activity of diffenent sized particles in Aristichthys nobilis and Salmo salar head soups. Food Fermentat Ind. (2019) 46:78–84. 10.13995/j.cnki.11-1802/ts.022206

[7] Wang H Gao G Ke L Zhou J Rao P Jin Y. Isolation of colloidal particles from porcine bone soup and their interaction with murine peritoneal macrophage. J Funct Foods. (2019) 54:403–11. 10.1016/j.jff.2019.01.021

[8] Wu D Ke L Liu H Lv W Liu S Zhou J. Antidiabetic effects of Ge-Gen-Qin-Lian-Tang decoction and its aggregated compositions on STZ-induced diabetic Wistar rats. J Fuzhou Univ. (2014) 42:957–62. 10.7631/issn.1000-2243.2014.06.0957

[9] Zhang J Tao N Qian X Wang X Wang M. Evaluation of antioxidative capacity and lipidomics profiling of big eye tuna (*Thunns obesus*) head soup with different colloidal particle size. Int J Food Sci Tech. (2020) 55:3254–66. 10.1111/ijfs.14588

[10] Lin L Tao N Su H Zhang J Zhong J. Migration of nutrients and formation of micro/nano-sized particles in Atlantic salmon (*Salmo salar*) and bighead carp (*Aristichthys nobilis*) head soups. Food Biosci. (2020) 36:100646. 10.1016/j.fbio.2020.100646

[11] Fan X Li X Tao N Zhang J Wang M Qian X. Effect of salt addition time on the nutritional profile of thunnus obesus head soup and the formation of micro/nano-sized particle structure. Molecules. (2019) 24:4447. 10.3390/molecules24244447 31817288 PMC6943628

[12] Zhai Z Fan X Tao N. Effect of salt concentration on the formation of micro/nano particles in tuna head soup. Food Sci. (2021) 42:21–9. 10.7506/spkx1002-6630-20200103-028

[13] Liang L Qi C Wang X Jin Q McClements D. Influence of homogenization and thermal processing on the gastrointestinal fate of bovine milk fat: *In vitro* digestion study. J Agr Food Chem. (2017) 65:11109–17. 10.1021/acs.jafc.7b04721 29124931

[14] Zhao L Du M Mao X. Change in interfacial properties of milk fat globules by homogenization and thermal processing plays a key role in their *in vitro* gastrointestinal digestion. Food Hydrocoll. (2019) 96:331–42. 10.1016/j.foodhyd.2019.05.034

[15] Minekus M Alminger M Alvito P Ballance S Bohn T Bourlieu C. A standardised static *in vitro* digestion method suitable for food – an international consensus. Food Funct. (2014) 5:1113–24. 10.1039/C3FO60702J 24803111

[16] Zhang R Zhang Z Zhang H Decker E McClements D. Influence of emulsifier type on gastrointestinal fate of oil-in-water emulsions containing anionic dietary fiber (pectin). Food Hydrocoll. (2015) 45:175–85. 10.1016/j.foodhyd.2014.11.020

[17] Yao H Liu W Lin L Ying L Gan J Liu Y. Micro-nano particle formation and transformation mechanisms of broth in meat braised processing. Food Chem. (2021) 342:128383. 10.1016/j.foodchem.2020.128383 33097328

[18] Folch J Lee M. Sloane-Stanley G. A simple method for the isolation and purification of total lipids from animal tissues. J Biol Chem. (1957) 226:497–509. 10.1016/S0021-9258(18)64849-5 13428781

[19] Zhang J Tao N Wang M Shi W Ye B Wang X. Characterization of phospholipids from pacific saury (*Cololabis saira*) viscera and their neuroprotective activity. Food Biosci. (2018) 24:120–6. 10.1016/j.fbio.2018.06.002

[20] Inguglia E Zhang Z Tiwari B Kerry J Burgess C. Salt reduction strategies in processed meat products – a review. Trends Food Sci Tech. (2017) 59:70–8. 10.1016/j.tifs.2016.10.016

[21] Hur S Decker E McClements D. Influence of initial emulsifier type on microstructural changes occurring in emulsified lipids during *in vitro* digestion. Food Chem. (2009) 114:253–62. 10.1016/j.foodchem.2008.09.069

[22] Sarkar A Goh K Singh H. Colloidal stability and interactions of milk-protein-stabilized emulsions in an artificial saliva. Food Hydrocoll. (2009) 23:1270–8. 10.1016/j.foodhyd.2008.09.008

[23] Gallier S Ye A Singh H. Structural changes of bovine milk fat globules during *in vitro* digestion. J Dairy Sci. (2012) 95:3579–92. 10.3168/jds.2011-5223 22720916

[24] Csorba S Nagy B Varga S Marodi L. Protective function of human milk. Acta Paediatr Acad Sci Hung. (1980) 21:93–105. 6970496

[25] Shimizu M Miyaji H Yamauchi K. Inhibition of lipolysis by milk fat globule membrane materials in model milk fat emulsion. Agric Biol Chem. (1982) 46:795–9. 10.1080/00021369.1982.10865129

[26] Ye A Cui J Singh H. Proteolysis of milk fat globule membrane proteins during *in vitro* gastric digestion of milk. J Dairy Sci. (2011) 94:2762–70. 10.3168/jds.2010-4099 21605745

[27] Hou S He S Xie J Li M Hong M Guan F. Integral characterization of normal and alopecic hair at different degeneration stages by in-situ visible and chemical imaging. Spectrochim Acta A. (2020) 235:118315. 10.1016/j.saa.2020.118315 32289732

